# Ocular ischemic syndrome secondary to carotid artery disease: a comprehensive review addressing critical early detection, management, and education

**DOI:** 10.3389/fopht.2026.1717841

**Published:** 2026-01-28

**Authors:** Eduardo Ávila-Figueroa, María Flores-Calvo, Christopher C. Sánchez-Rodríguez, Irving A. Domínguez-Varela, Patricia Salazar-Ramírez, Fernanda Zamora Cortina, Félix E. Tena-Betancourt, Javier E. Anaya-Ayala

**Affiliations:** 1 Facultad Mexicana de Medicina, Universidad La Salle, Mexico City, Mexico; 2 ENT Department, Instituto Nacional de Rehabilitación Luis Guillermo Ibarra Ibarra, Mexico City, Mexico; 3 Internal Medicine Department, Hospital Ángeles del Pedregal, Mexico City, Mexico; 4 Cardiology Department, Instituto Nacional de Cardiología Ignacio Chavez, Mexico City, Mexico; 5 Department of Ophthalmology, Asociación Para Evitar la Ceguera (APEC), Hospital de la Ceguera, Mexico City, Mexico; 6 Universidad Autónoma de San Luis Potosí, San Luis Potosí, Mexico; 7 Community Health and Epidemiology, University of Saskatchewan, Saskatoon, SK, Canada; 8 Department of Surgery, Section of Vascular Surgery and Endovascular Therapy, Instituto Nacional de Ciencias Médicas y Nutrición Salvador Zubiran, Mexico City, Mexico; 9 Dirección Médica Hospital Ángeles Universidad, Mexico City, Mexico

**Keywords:** atherosclerotic carotid disease, detection and management, education, occlusive disease, ocular ischemic syndrome

## Abstract

**Purpose:**

This study was conducted to emphasize the significance of early detection of ocular ischemic syndrome (OIS), indicating advanced carotid atherosclerotic occlusive disease and preceding life-threatening ischemic cerebrovascular events, and to boost education in eyesight care.

**Methods:**

A systematic literature search was performed using PubMed and Embase databases using the keywords “carotid disease,” “ocular ischemic syndrome,” “amaurosis fugax,” “early detection,” and education, selecting 45 articles containing relevant information for our focused review.

**Results:**

OIS is a sight-threatening condition caused by ocular hypoperfusion, secondary to occlusive disease of the common or internal carotid arteries. We observed that this condition can be associated with multiple clinical manifestations, such as progressive or acute visual loss, orbital pain, and iris neovascularization. Carotid artery disease is frequently underdiagnosed due to an unfocused clinical examination of the eye fundus, the presence of carotid bruit, and other similarities associated with ocular ischemic syndrome, while the management of this condition should always be carried out with a multidisciplinary approach by implementing techniques such as panretinal photocoagulation (PRP), the use of ophthalmologic drugs, and surgical management of carotid via endarterectomy.

**Conclusions:**

Among clinicians, OIS is frequently undetected because the range of symptoms initially exhibited by an individual can mimic age-related visual decline, delaying detection until serious complications such as amaurosis fugax or stroke occur. Proficient skills in fundus examination and carotid artery auscultation are crucial in identifying this harmful condition, including the use of non-invasive procedures and full recognition of the need for early eye care education.

## Introduction

**Figure 1:**
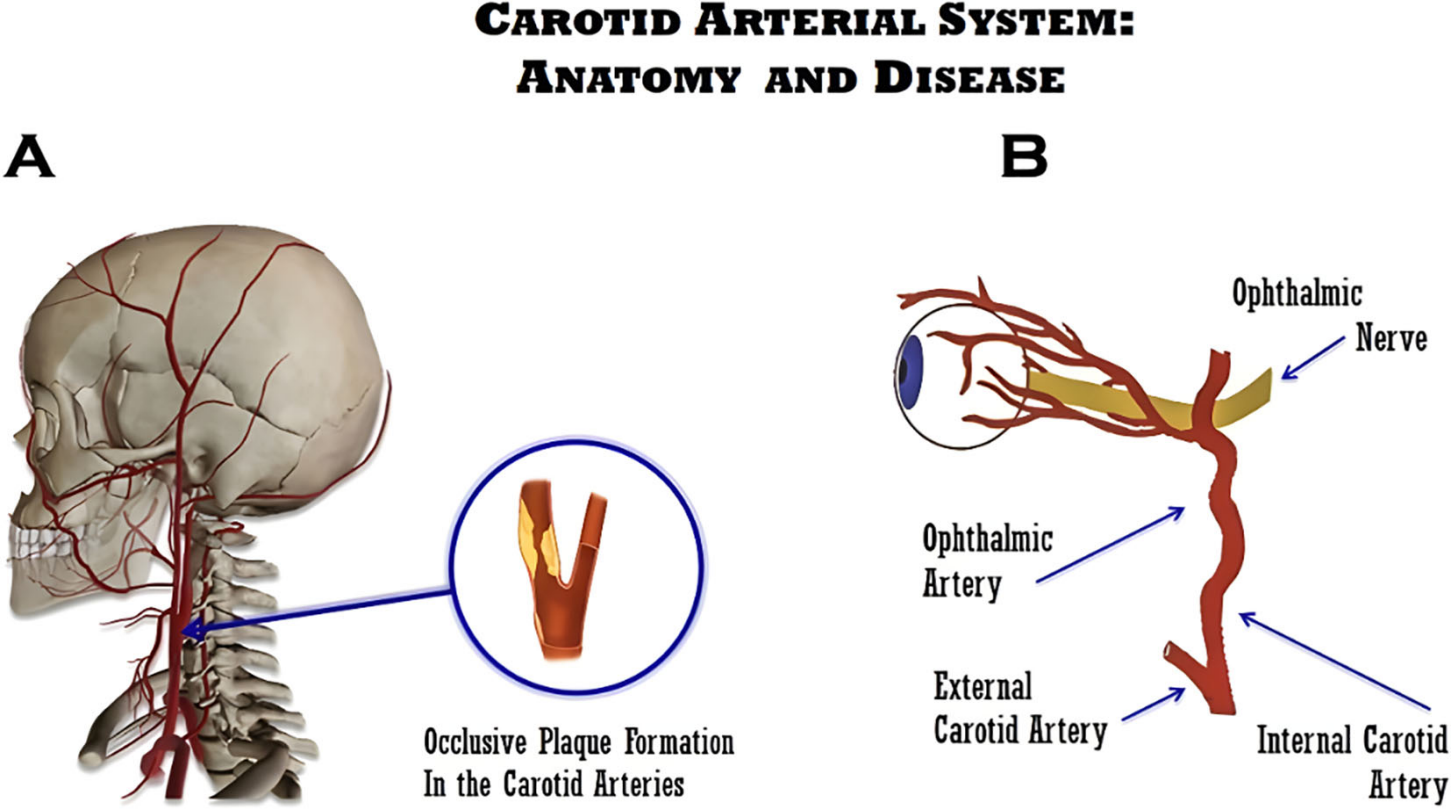
Schematic illustrations of carotid disease. **(A)** Illustration of the carotid atherosclerotic arterial disease. **(B)** Relationship between the internal carotid artery (ICA) and the ophthalmic artery, which explains the ocular ischemic syndrome (OIS) and its associated symptoms.

The insidious progression of carotid artery disease (CAD) is noted as a global health burden, associated with substantial socioeconomic impact. Its development occurs through the narrowing of the common, internal, or external carotid arteries, responsible for supplying oxygen-rich blood to the brain (1). The reduction in arterial lumen is primarily caused by the presence of atherosclerotic plaque buildup, which over time thickens and hardens the arteries (2). The 2021 European Guidelines on Cardiovascular Disease Prevention in Clinical Practice have defined plaque as the presence of focal wall thickening, seen on carotid ultrasound as ≥50% greater than the surrounding vessel wall, or as a focalized region with an intima–media thickness measurement of ≥1.5 mm, protruding into the lumen (3). Atherosclerotic disease is a systemic vascular condition that can occur in any artery throughout the body (4), although arterial obstruction can result also from a variety of causes, as external compression, arterial dissection, dissecting aneurysms of the carotid vessels, and vasculitis (such as Takayasu arteritis, giant cell arteritis, aortic arch syndrome, and Behçet’s disease), including trauma or inflammation (**Figures 1A, B**). However, the most common cause of chronic inadequate vascular supply to the ophthalmic arteries is atheromatous disease of the carotid artery (5–7). The ophthalmic artery is the first intradural branch of the carotid artery, while its activity is reflected in proper ocular microcirculation and carotid artery blood flow. Kearns and Hollenhorst were among the first to report this condition in 1963, by describing a patient with ocular symptoms and signs associated with severe carotid artery obstruction—a condition referred to as “venous stasis retinopathy” (8), affecting approximately 5% of patients with severe carotid artery insufficiency or thrombosis. Over time, such term elicited confusion since it was also used to describe mild central retinal venous occlusion (9), thus leading to the term ocular ischemic syndrome (OIS), coined to address clinical features of the eye and vision syndromes associated with chronic carotid artery obstruction (10) (**Figures 1A, B**). Remarkably, CAD progresses without noticeable symptoms for years, showing no symptoms until reduced blood flow or rupture of plaques occurs, causing tissue hypoxia. Nevertheless, there have been cases where OIS was elicited by internal carotid artery (ICA) dissection (11). Among chronic conditions, embolic retinopathies, light-induced amaurosis, or neovascular glaucoma can appear. Acute events that occur can lead to sudden vision loss, embolic stroke, transient ischemic attack (TIA), and retinal artery occlusion (12). During physical examination, most of these ophthalmic signs can be observed throughout fundoscopy, properly correlated with substantial carotid artery stenosis (13). Patients suffering from carotid artery stenosis typically develop carotid bruit, which can be heard as a result of the turbulent flow occurring within the narrowed artery (3, 14).

Consequently, duplex ultrasound (DUS) is the first-line imaging modality to assess vessel anatomy and the presence of blockages or stenosis. Under such assertion, the European Carotid Surgery Trial (ECST) and the North American Symptomatic Carotid Endarterectomy Trial (NASCET) guidelines rely on the luminal diameter to assess the severity of the carotid artery stenosis (15). Ocular ischemic syndrome is frequently misdiagnosed due to overlapping features with other retinal vascular disorders, such as central retinal vein occlusion and diabetic retinopathy. However, OIS is distinguished by chronic hypoperfusion, mid-peripheral dot-blot hemorrhages, delayed choroidal filling on fluorescein angiography, anterior segment ischemia, and ocular or periorbital pain, which are less typical of other occlusive diseases. The aim of this work was to describe the impact of risk factors and clinical manifestations falling under the OIS syndrome, outlining non-invasive methods to reach a presumptive diagnosis, while our secondary goal was to emphasize the importance of early carotid artery diagnosis to limit delays associated with severe complications and disabilities while disclosing the relevance of promoting early eye care among young adult individuals.

## Methods

A systematic literature narrative review using the PubMed and Embase databases was conducted for this study. Keywords such as “carotid disease”, “ocular ischemic syndrome”, “amaurosis fugax”, and “early detection” were used to select relevant articles published up to January 2025, resulting in 114 articles. The initial search was performed in January 2024, with the final update completed in January 2025. Comprehensive reviews and clinical studies relevant to the topic were included in this review to assess the abstracts and full texts of articles, eliminating duplicates and excluding papers written in languages other than Spanish or English. Only 45 articles were selected for this review, and there were no limitations in doing this work. Two investigators independently screened the titles and abstracts, followed by full-text review according to predefined inclusion criteria.

## Historical background

Ocular ischemic syndrome is a complex condition that predominantly results from ocular hypoperfusion due to stenosis or occlusion of the common or internal carotid arteries, with chronic atherosclerosis as the major cause of carotid changes. Early accounts by Hedges in 1963 described how a complete obstruction of the left internal carotid artery led to the formation of peripheral blot hemorrhages, dilating retinal veins, a serious condition associated with retinal hypoxia due to carotid artery stenosis (1). Similarly, Kearns and Hollenhorst reported analogous ocular symptoms associated with carotid artery stenosis, further naming it as venous stasis retinopathy, which was seen in a small segment of patients (2). Over the years, various terms have been used to characterize ocular changes secondary to carotid artery stenosis, predominantly analogous to ischemic ophthalmopathies, leading to damage of the optic nerve and other eye tissues, resulting in visual problems such as vision loss or visual field changes. Finally, since the signs of ischemia were prevalent in both the anterior and the posterior segments of the eye, it led to the coinage of the contemporary term ischemic syndrome characterized by carotid artery occlusive disease (2).

## Epidemiology

Ocular ischemic syndrome is a complex disease, mostly affecting the elderly with a mean age of 65 years or older. Although its current prevalence remains undetermined, it is rarely seen in individuals under 50 years old. The syndrome is more prevalent in populations with a high burden of atherosclerotic disease, with reported associations across North America, Europe, and Asia (16). Early figures reported by Sturrock and Mueller in 1984 suggested an incidence of 7.5 cases per million people per year (17), although these were considered underestimated since OIS is frequently misdiagnosed as central retinal vein occlusion (CRVO) or diabetic retinopathy, with men being affected twice as often as women (18). This syndrome is associated with various symptoms, the most common being gradual vision loss over weeks or months, as reported in 80% of cases (17). Notably, up to 29% of patients with asymptomatic carotid disease exhibit retinal vascular changes, and approximately 1.5% of these symptoms progress to symptomatic OIS each year (19).

Carotid atherosclerosis is considered the main cause of OIS; however, any condition that reduces the lumen of the carotid artery can lead to hypoperfusion and result in ischemia. One notable population-based study by Song P. et al. (16) reported an alarming increase of the carotid intima–media thickness among patients aged 30 to 79 years old, estimated at 27.6% (95% CI 16.9–41.3); carotid plaque at 21.1% (95% CI 13.2–31.5); and carotid stenosis at 1.5% (95% CI 1.1–2.1), findings compatible with a notorious global increase bordering above 50% in all cases. Even though the global prevalence of carotid stenosis is consistently lower in women than men of all ages (30 to 79 years), it occurs in asymptomatic people secondary to increased carotid intima–media thickness and insidious carotid plaque (16). The risk factors associated with the increase of carotid intima–media thickness include smoking, type 2 diabetes, and hypertension. The same report referred to this population as high risk for carotid plaque formation (2). On the other hand, high HDL levels were considered a protective factor for carotid plaque, and in women, these were associated with reduced likelihood of both plaque and increased carotid intima–media thickness. The cardinal risk factors for both conditions are described in **Table 1** (16).

**Table 1:** Risk factors associated with carotid disease occurrence and their statistical significance (16).

Risk factor	OR	95% CI	*P*-value
Age (>50 years)	2.71	14.1–30.5	<0.001
Smoking	1.76	1.43–2.30	<0.001
Diabetes mellitus	2.23	1.48–3.36	<0.001
Systemic hypertension	1.55	1.03–2.34	0.038
Gender (female)	0.49	0.38–0.63	<0.001
HDL levels	0.46	0.21–0.99	0.048

Cardinal factors predisposing to carotid artery disease. Age is noted as a significant factor, with risk increasing with age.

OR, odds ratio; CI, confidence interval; HDL, high-density lipoprotein.

## Risk factors for ocular ischemic syndrome

OIS is most frequently observed in older adults and represents a downstream manifestation of advanced carotid atherosclerotic disease. Age is the most significant non-modifiable risk factor, with a mean age of presentation of approximately 65 years and a rare occurrence before the age of 50 (2). A global systematic review and meta-analysis demonstrated a consistent increase in the prevalence of increased carotid intima–media thickness, carotid plaque, and carotid stenosis with advancing age across all regions, particularly after the fifth decade of life (16). As carotid atherosclerosis progresses, ischemic injury characteristic of OIS occurs in both the anterior and posterior segment structures.

In addition to age, several systemic cardiovascular risk factors play a central role in the development and progression of OIS. Hypertension, diabetes mellitus, and smoking are commonly present in patients with OIS and contribute to accelerated carotid plaque formation through endothelial injury, inflammation, and metabolic dysregulation (2). Male sex has also been associated with a higher prevalence of carotid atherosclerosis, whereas higher high-density lipoprotein (HDL) cholesterol levels appear to have a protective effect against plaque development (16).

## Pathophysiologic data

OIS primarily develops in patients suffering from poor collateral circulation between the internal and external carotid arteries or between the two internal carotid arteries, where stenosis of less than 50% of the ICA may suffice to lead to symptomatic OIS. Patients with well-developed collateral circulation may not develop symptoms, even when the ICA is partially occluded. During funduscopic examination, emboli were a common finding in nearly half of the cases of branch retinal artery obstruction; in cases where emboli were not clinically visible, fundus autofluorescence (FAF) enhanced their visualization, with emboli composed mainly of cholesterol and calcium (20, 21). In OIS patients suffering from stenosis involving ≥90% of the common or internal carotid arteries, a usual finding was seen on the affected side, while in 50% of cases, the affected artery was completely obstructed (10). This occurred because stenosis reduced by half the perfusion pressure of the central retinal artery (22). In addition, OIS patients can exhibit reduced blood flow of retrobulbar vessels and reverse blood flow of the ophthalmic artery (OA) (5), forcing the artery to shunt blood away from the eye to a low-resistance intracranial circuit, worsening a reduction of retrobulbar blood flow, which concomitantly leads to hypoperfusion and ischemia of the ocular tissues (5). Typically, the atherosclerotic plaque consists of extracellular fat particles and foam cells accumulating within the intima of the arterial wall, forming a lipid or necrotic core, which is surrounded by a collagen matrix and smooth muscle cells, and having an endothelial layer covering the outer surface, known as the fibrous cap. Subsequently, inflammatory cells, primarily T cells and macrophages, infiltrate the strata of such lesions, leading to thrombosis and potential plaque rupture (23). Plaque rupture and erosion are often caused by fissures of the fibrous cap, thus exposing the core to blood or endothelial damage, leading to thrombosis without cap rupture. By its own nature, a vulnerable plaque has a thin fibrous cap (<65 μm) with activated macrophages that, within a prothrombotic environment, can result in thrombus formation, which may cause strokes or heart attacks (24, 25).

## Clinical manifestations and findings

Gathering relevant information is essential to assess the level of acuity of a patient’s complaint and narrow down a differential diagnosis. Such an approach includes data on the severity of the complaint, laterality, onset, course, duration, and history of previous analogous episodes, history of eyeglass use, and chronic eye diseases such as glaucoma or macular degeneration. It is also important to inquire about other associated symptoms, such as nausea, vomiting, headache, fever, focal weakness, paresthesia, joint or muscle pain, headache, auras preceding migraines, flashes of light or floaters, and double vision (26). All clinical manifestations of OIS result from tissue hypoxia. The physician must obtain a comprehensive clinical history to identify signs and symptoms related to this syndrome, such as periorbital pain, changes in the visual field, loss of vision, amaurosis fugax, and light-induced amaurosis (12, 27).

The loss of vision of the affected eye is present in over 90% of patients affected with OIS (14), from which 67% suffered this loss gradually over a period of weeks to months, 12% happened over a period of days, and another 12% occurred acutely and happening within seconds or minutes. On the other hand, 10% of patients also present AF, usually resulting from a transient embolization occurring at the central retinal artery (5, 10). In patients with light-induced amaurosis, recovery of visual function after exposure to bright light would depend on the severity of carotid stenosis (22). In recently diagnosed OIS patients, visual acuity is assessed using the Snellen chart and ranges from 0.4 to 1.0 in 43% and counting fingers or worse in 37% (14). The visual fields of patients with OIS can vary from normal to central scotoma, centrocecal defects, nasal defects, or the presence of only a temporal or central island (27); 40% of patients may also complain of pain in the affected eye, or even around the periorbital area, which could be secondary to neovascular glaucoma or hypoxia of the dura mater or eyeball. The pain is commonly described as dull with gradual development and relieved when lying down (27).

**Figure 2:**
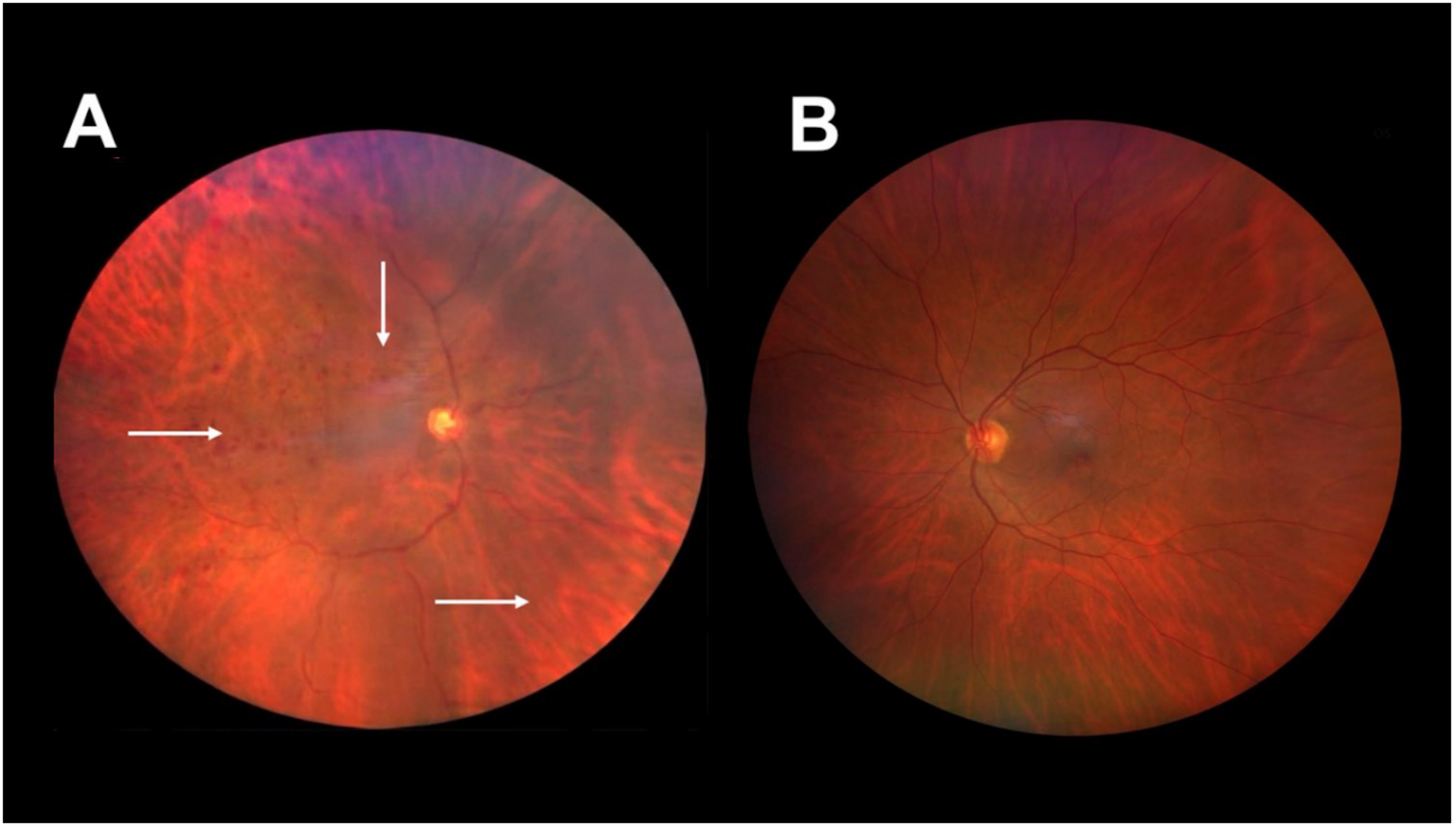
Fundus image of the right and left eyes of an 50-year-old man with a diagnosis of hypertension, dyslipidemia, 90% ipsilateral carotid artery obstruction, and ischemic ocular syndrome in the right eye. **(A)** Right eye with intraretinal hemorrhages in all four quadrants (arrows), with greater distribution in the mid-periphery and an altered artery-to-vein ratio due to dilated retinal veins. **(B)** Right eye with no evidence of ischemic ocular syndrome. Image courtesy of Hospital Ángeles Universidad, Surgery Department. Used with permission.

When subjected to a fundoscopy exam, typically nearly 66% of patients with sudden visual loss show neovascularization of the iris and iridocorneal angle (22). This condition, known as neovascular glaucoma, occurs due to the formation of new blood vessels blocking the outflow of aqueous humor from the eye, increasing intraocular pressure (IOP) in 50% of the affected patients. Loss of light perception is usually developed during later stages (27). Other ocular manifestations may include a cherry-red spot in 12% of cases, related to embolization of the retinal artery and opalescence of the aqueous humor occurring in almost 20% of cases, where inflammatory cells are also observed at the anterior chamber, and possible opacity of the lens, along with a fixed or semi-dilated pupil, due to atrophy and ischemia of the ciliary muscle (5, 22). Other signs of OIS might include corneal edema, dilation of the conjunctival and episcleral vessels, and in the rarest cases, liquefactive necrosis of the cornea (5, 28). When examining the posterior chamber, retinal arteries appear narrowed, while veins appear dilated. Retinal pulsations and hemorrhages may be typically observed, located in the external retinal layers at the mid-periphery, not confluent, and could be present in 80% of patients (**Figure 2**). Microaneurysms may also be seen at the macula and the mid-periphery often accompanied by telangiectasias (5, 14). The funduscopic exam should be a critical component for every physical examination intended to assess the eye fundus with an ophthalmoscope; it allows examining the inner structures of the eye, particularly the fundus, which includes the retina, optic nerve, and blood vessels. While the technical aspects of ophthalmoscopy are well established, its diagnostic value in OIS lies in the recognition and interpretation of specific ischemic patterns.

**Figure 3:**
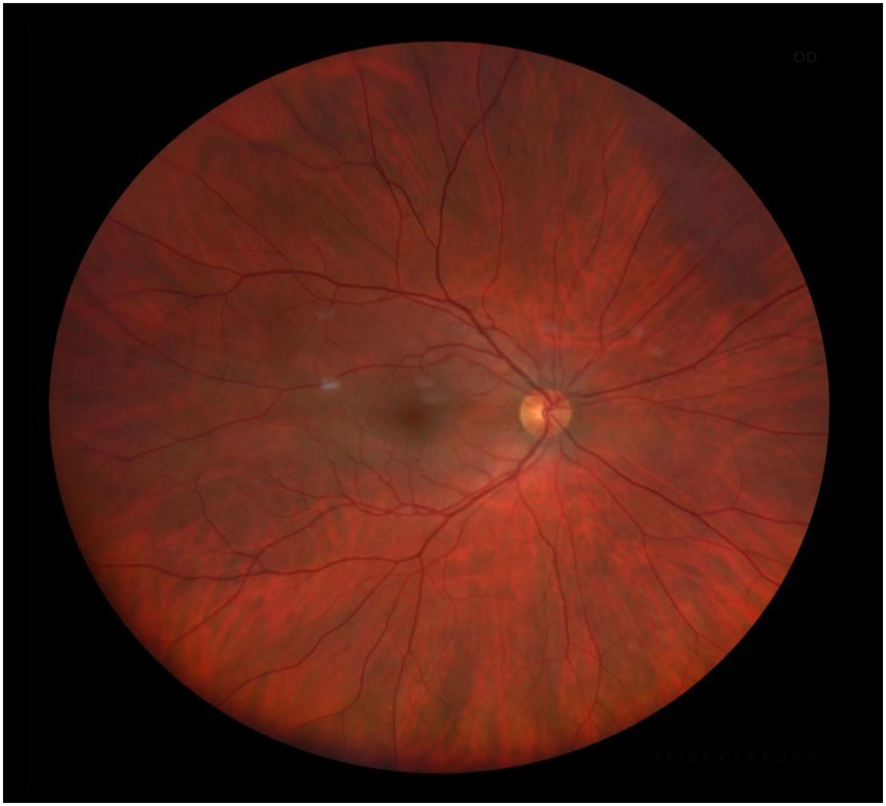
Fundus image of the right eye of a male patient. Optic nerve excavation with normal characteristics; normal artery–vein relationship with no alteration in the middle periphery or macular region.

In patients with suspected OIS, careful evaluation of the optic disc and surrounding retina is essential. The optic disc should be assessed for ischemic changes and asymmetry when compared with the contralateral eye. In a healthy patient, it appears yellow-pink, contrasting with the red, brown, or orange hues of the surrounding retina (**Figure 3**). The disc is sharply demarcated temporally and, to a lesser extent, nasally from the background retina, which includes all areas that are not occupied by the disc, blood vessels, or macula. A stepwise funduscopic exam procedure is outlined in **Table 2** (29).

**Table 2:** Routine, stepwise funduscopic examination of the eyes describing comprehensive key steps, from positioning and adjusting the ophthalmoscope to observing the fundus and documenting clinical findings (29).

Brief description of the action plan	
1.	Asking patients to remove their eyeglasses but not their contact lenses.
2.	Lowering the room lights should be done by the examiner. When standing in front of patients, the examiner should direct a spot of light directly in front of them to fix their gaze.
3.	Holding the ophthalmoscope to see well through the patient’s eye and then switching the light at two-thirds of strength.
4.	Standing 15° temporal to the patient’s optical axis, and with an eye-to-eye distance of 30 cm, setting the lens wheel at +10 diopters.
5.	Placing the contralateral palm on the patient’s forehead, with the examiner’s thumb on the patient’s supraorbital ridge, to hold their head to prevent accidentally bumping with the instrument.
6.	Slowly moving toward the patient to gradually decrease diopters toward zero to increase focal length. In this way, focusing successively on the cornea, lens, vitreous, and finally the retina.
7.	Getting as close as 3–5 cm with the instrument from the patient’s eye; when seeing the retina, look for the optic disc or a vessel; bring the structure into sharp focus by rotating the lens wheel as needed. In this case, 0 reading often works well. Note that myopic examiners may need a negative number instead.
8.	Moving along the vessel toward a larger caliber until the optic disc is reached, the examiner must study color, lateral margins, and the size of the optic cup; when the disc shows an elevation, the pattern of vessels emerging from it and the pulsations of the retinal veins overlying the disc are also evaluated.
9.	Returning to the disc by following the vein course.
10.	Repeating the procedure in the three other quadrants.
11.	Finally, directing the beam temporally, and asking the patient to look at the light.
12.	Switching the ophthalmoscope to the other hand and eye to repeat the procedure.

Visual function testing complements funduscopic findings and aids in narrowing the differential diagnosis. Visual acuity should be assessed in each eye, and confrontation visual field testing may reveal asymmetric or focal defects suggestive of retinal or optic nerve ischemia (30). Carotid auscultation is then done by placing the bell of the stethoscope over each carotid artery while listening for a soft, high-pitched “swooshing” sound.

## Diagnosis

Early detection of OIS is critical, as ocular manifestations may precede catastrophic cerebrovascular events. A comprehensive medical assessment should consider the assessment of both entities, OIS and CAD. The most specific feature of OIS is delayed choroidal filling time, considered a specific angiographic sign with a patchy pattern, which can be observed in 60% of cases detected through fluorescein angiography. Delay in choroidal filling as well as prominent arterial staining is usually absent in central retinal vein occlusion, considered to be important in the main differential diagnoses. This marker is the most important modality to diagnose OIS syndrome. The filling delay time can range from 10 to 12 s or even more than a minute from the first appearance of the dye until a complete choroidal filling can be observed. A significant prolonged arteriovenous time is critical when transit takes longer than 11 s from the first appearance of retinal arterial dye until complete retinal venous filling occurs, considered the most sensitive parameter in 95% of cases.

**Figure 4:**
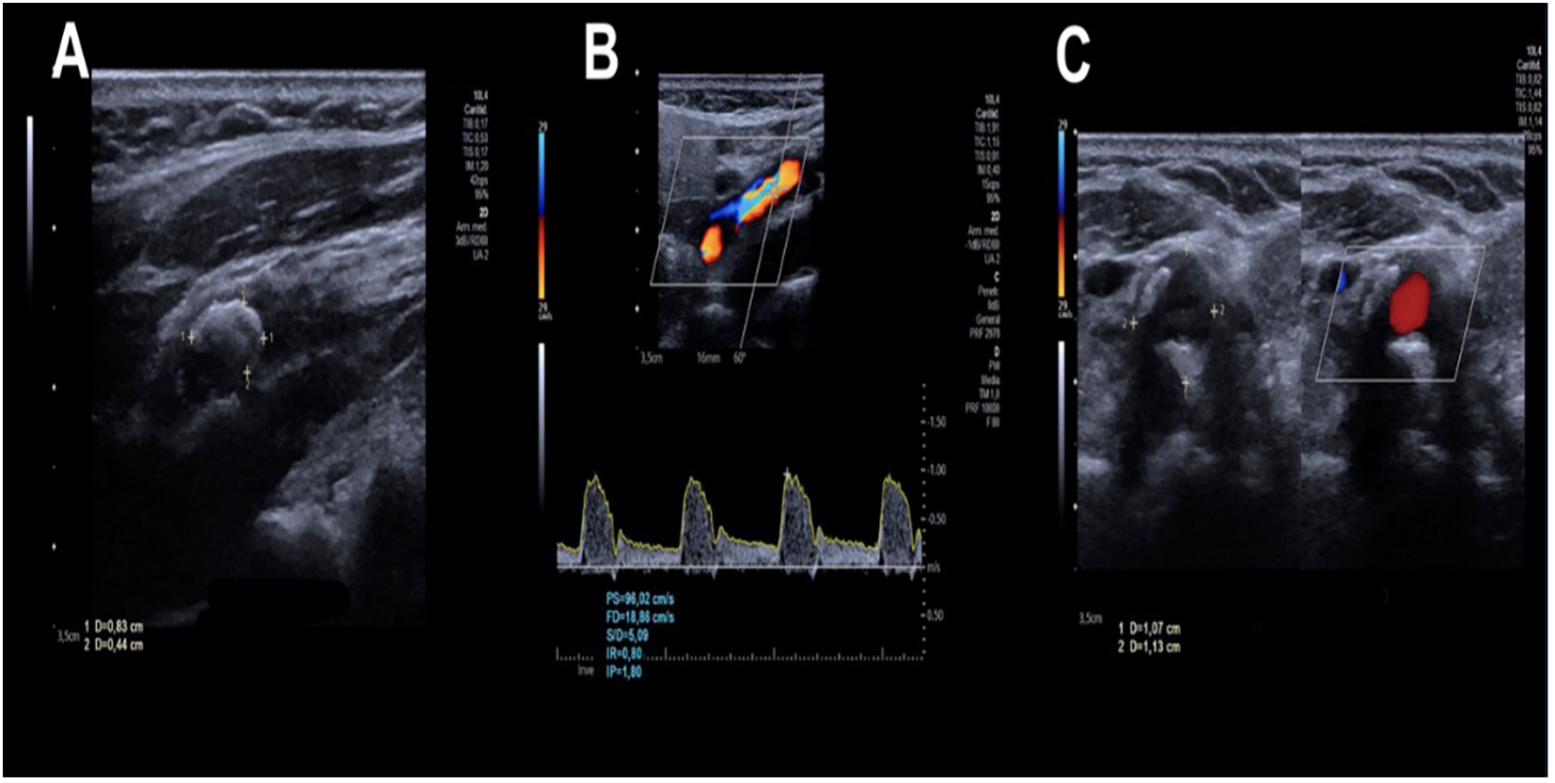
A carotid duplex ultrasound study of the right carotid artery of an 82-year-old patient was performed using real-time equipment with a multifrequency linear transducer in grayscale, color, and spectral Doppler. **(A)** At the level of the bulb, there is a plaque adjacent to the anterior and posterior wall, calcified, with extension toward the proximal segment of the internal carotid artery, measuring 9 mm and causing a significant decrease in vascular diameter. **(B)** Parameters measured at the level of stenosis: peak systolic (PS), diastolic flow (DF), systole/diastole ratio (S/D), and resistance indices (RIs). **(C)** Adjacent plaque to the posterior wall, calcified, measuring 10 mm and causing a significant decrease in vascular diameter. Image courtesy of Hospital Ángeles Universidad, Surgery Department. Used with permission.

Nonetheless, this parameter is somewhat considered the least specific (10). It has been observed that 85% of cases can present late arterial staining due to endothelial damage secondary to ischemia. Macular edema occurring in the later phases of the angiogram is seen in 17% of cases. Other signs may include retinal capillary non-perfusion, hyperfluorescence of microaneurysms, and hyperfluorescence of the optic nerve head (31). DUS is an accessible non-invasive imaging method having a sensitivity of 94% and a specificity of 92% when diagnosing 60%–99% of carotid stenosis (32). According to the Clinical Practice Guidelines of the European Society of Vascular Surgeons for Management of Atherosclerotic Carotid and Vertebral Artery disease, 2023 edition (33), due to its low cost and accessibility, DUS is usually the first-line imaging modality to diagnose carotid arterial disease (34). B-mode imaging is combined with color flow and the ability to undertake Doppler flow velocity measurements. Peak systolic (PS), diastolic flow (DF), and systole/diastole ratio (S/D) are used to quantify the severity of carotid stenosis, while resistance indices (RIs) reflect vascular resistance and hemodynamic compromise (35) (**Figure 4**). Computed angiotomography (CTA) and MR angiography (MRA) may also be successful for diagnosis, having the advantage as well showing the aortic arch, supra-aortic trunks, carotid bifurcation, and the distal internal carotid artery. While DUS is widely accessible and non-invasive, it is operator-dependent and may underestimate complex or heavily calcified lesions. On the other hand, CTA provides good spatial resolution but involves radiation and contrast exposure. MRA avoids ionizing radiation but may overestimate stenosis and is less accessible. A combination of DUS + CTA or MRA provides dynamic or functional information about eye blood flow and diagnostic accuracy (34). Intra-arterial angiography is no longer in use because of the risk of angiography-related strokes (15). Routine ultrasound screening for asymptomatic carotid stenosis is not recommended for the general population, unless considered for patients with multiple risk factors to reduce morbidity and mortality, optimize medical therapy, and guide risk stratification, rather than immediately carrying out carotid interventions. Retinal or visual acuity changes suggest that OIS is mostly linked to carotid stenosis, often due to chronic hypoperfusion (5).

## Management

The management of OIS requires the identification of three main factors: first, recognizing various eye conditions and their associated or deleterious symptoms, which can range from minor discomfort to severe vision loss; second, treating the associated systemic causative factors and associated comorbidities; and third, performing any surgical intervention as required, establishing a prior multidisciplinary approach (12). Panretinal photocoagulation (PRP) helps to reduce oxygen demand in the ischemic retina, decreasing the risk of neovascular glaucoma, although its effectiveness may be limited by VEGF production in other ocular areas. On the other hand, using intravitreal anti-VEGF therapy can control both neovascularization and associated macular edema. When intraocular pressure does not respond to medical treatment, trabeculectomy may be indicated with anti-VEGF administered beforehand, since it plays a crucial role in the growth and development of blood vessels to reduce the risk of postoperative hemorrhage. When treating the anterior chamber inflammation, topical steroids and mydriatics can be highly effective (9) while preventing temporary vision changes; pro-inflammatory agents such as prostaglandins and pilocarpine should be avoided (36).

Drugs that reduce aqueous production, such as β-blockers and α-agonists, may be used to reduce increased IOP. Prostaglandin analogs, pilocarpine, and anticholinergic drugs should be avoided to prevent inflammation (37). Panretinal laser photocoagulation may be considered for people with OIS who have iris neovascularization and/or retinal neovascularization (38). Alternatively, the effective treatment of OIS requires a multidisciplinary approach; that is, besides the ophthalmologic evaluation, patients with OIS should be thoroughly evaluated by a neurologist and a general physician to characterize and treat associated and/or predisposing comorbidities. Surgical management will only benefit a patient visually if performed before the development of neovascularization of the iris and glaucoma occurs, as these markers indicate chronicity and irreversible damage. Carotid artery endarterectomy (CEA) and aspirin therapy (2-year stroke rate, 9%) showed better results in preventing stroke versus aspirin alone (2-year stroke rate, 26%) in patients with symptomatic or asymptomatic carotid artery stenosis (39). In asymptomatic cases, CEA is performed only when carotid artery stenosis reaches 60% to 99% (40). This surgery has proven to increase blood flow to the ophthalmic artery and prevent or reverse ischemic changes (37). An increase in IOP must be taken into consideration after ipsilateral CEA (41). The article by Kawaguchi et al. (42) discussed the effects of carotid artery bypass grafting on ocular circulation and chronic ocular ischemia syndrome in patients with carotid artery stenosis. CEA and carotid artery stenting (CAS) have been evaluated as an alternate treatment in high-risk patients. CEA involves surgical removal of atherosclerotic plaque from the carotid artery, whereas CAS is a less invasive approach in which a stent is placed to maintain luminal patency (38, 42). Post-surgery showed improvement of flow velocity in the ophthalmic artery (OphAr) and central retinal artery (CRA); sound results sustained throughout a 3-month follow-up demonstrated that this surgery corrected reversed flow in the OphA after increasing ocular flow, also indicating improvement in ocular hemodynamics. Clinically, 60% of patients with chronic OIS showed better visual acuity and less recurrent symptoms such as amaurosis fugax, suggesting that carotid revascularization may be effective in reversing some of the effects of ocular ischemia. This study supports surgery as treatment for OIS (42, 43).

## Discussion

Ocular ischemic syndrome can result in visual loss when carotid artery disease occurs; early stages of the disease may not cause perceptible symptoms despite the presence of atherosclerotic plaque of the carotid artery, although previous reports have shown figures of progression from 4% to 29% among other complexities surrounding this condition (44, 45). An in-depth analysis of our data demonstrated that the linking of CAD to clinical manifestations of OIS can be traced back over a decade ago. Nevertheless, when signs and symptoms are present, ocular manifestations can occur before cerebrovascular clinical presentation, which is why ophthalmologists and primary care physicians play a vital role in diagnosing and risk stratification of affected patients. Consequently, referring patients to a vascular surgeon, cardiologist, and a neurologist is critical to generate a comprehensive management of CAD and control potential life-threatening sequelae. Such gap tended to create a lack of awareness concerning the relationships between these conditions resulting in inadequate early clinical evaluation and late referrals, leading to vision impairment that significantly impacts the quality of life of individuals and increases their risk of later severe complications. More data on the incidence and prevalence of ocular ischemic syndrome are needed. As previously mentioned, if epidemiological information available dates back decades limiting our current understanding of this condition, it definitively poses one question: If the incidence of atherosclerotic disease has indeed increased, why would not ischemic eye syndrome also increase accordingly? Possible explanations include underdiagnosis, misclassification as other retinal vascular disorders, limited awareness among clinicians, and lack of contemporary epidemiological studies specifically addressing OIS. OIS displays multiple signs that can be seen on the eye fundus, such as a cherry-red spot, retinal neovascularization, increased IOP, macular microaneurysms, retinal vein pulsations, and neovascular glaucoma. Outside the eye fundus, signs like lens opacity, fixed pupils, corneal edema, dilation of conjunctival and episcleral vessels, and even liquefactive corneal necrosis may be visible. Symptoms may include visual loss, loss of light perception, AF, TIA, and alteration in visual fields, and periorbital pain may be also present.

The variety of presentations in ophthalmologic examinations may contribute to misdiagnosis of this condition, since distinguishing OIS from its differential diagnoses may be challenging. Early detection of OIS may be improved by heightened clinical suspicion in elderly patients with systemic atherosclerotic risk factors, asymmetric retinopathy, or unexplained ocular pain. Interdisciplinary collaboration between ophthalmologists, neurologists, and vascular specialists, along with routine consideration of carotid artery evaluation, may significantly reduce diagnostic delay. In this review, we highlighted the significance of proper eye fundoscopy and carotid auscultation, targeting also the clinical assessment for OIS due to CAD. Ocular findings are considered the first clinical manifestation of severe atherosclerotic plaque deposits of the carotid artery, which requires urgent intervention to prevent acute cerebrovascular disease, given the enormous risk of associated ischemic heart and neurological events. It is also worth mentioning that symptoms of OIS can resemble age-related visual loss, a common finding usually unnoticed for an extended period of time, until more serious events such as amaurosis fugax or stroke are present. Such events require a solid fundus examination and carotid auscultation to identify potential clinical changes. With these non-invasive and cost-effective methods, every physician should perform them to assess a presumptive diagnosis. Also, although DUS is clearly an effective non-invasive method to diagnose peripheral arterial disease (PAD) with a reliable scoring system for arterial mapping, it is not recommended as the first diagnostic choice due to its higher cost (46). This paper has emphasized not only the importance of early diagnosis and prior effective clinical screening to ensure that patients could be appropriately referred for the treatment of potential complications but also disclosing the need to increase medical awareness and risks involved in visual impairment though implementation of individual vision care education programs focusing on patients over 50 years of age or more prone to suffer insidious CAD. Overall, such an endeavor should include one-on-one counseling and access to assistive technologies for the proper assessment of visual functions, allowing physicians and related professionals to determine the best approach for maintaining healthy vision and preventing serious complications.

## Future perspectives

Future research in ocular ischemic syndrome should focus on the identification of early molecular and imaging biomarkers that enable timely diagnosis before irreversible tissue damage occurs. Advances in multimodal retinal imaging, combined with molecular profiling of oxidative and inflammatory pathways, may facilitate risk stratification and personalized management. Furthermore, the development of targeted therapies aimed at modulating oxidative stress, inflammation, and regulated cell death pathways represents a promising avenue to improve visual outcomes in OIS.

## Limitations

This review has several limitations. First, it was designed as a narrative review rather than a systematic review, which may introduce selection bias. Second, only two scientific databases were used for the bibliographic search, potentially limiting the completeness of the included literature. Additionally, heterogeneity among available studies and the relative scarcity of large prospective clinical trials in ocular ischemic syndrome restrict the strength of some conclusions.
